# An Assessment of the Subjective Psychological and Social Effects of Malocclusion-Related Dental Aesthetics and Its Influence on Body Self-Image and Oral Health-Related Quality of Life in Young Adults

**DOI:** 10.7759/cureus.60120

**Published:** 2024-05-11

**Authors:** JS Yamini Priyanka, Palavalli Bhavya, Baratam Srinivas, Gowri Sankar Singaraju, Ganugapanta Vivek Reddy, Prasad Mandava

**Affiliations:** 1 Orthodontics and Dentofacial Orthopedics, Vydehi Institute of Dental Sciences, Bangalore, IND; 2 Orthodontics and Dentofacial Orthopedics, Narayana Dental College, Nellore, IND; 3 Orthodontics and Dentofacial Orthopedics, Anil Neerukonda Institute of Dental Sciences, Visakhapatnam, IND

**Keywords:** ohrqol, pidaq, ohip, orthodontic, malocclusion

## Abstract

Introduction: Malocclusion has a psychological impact related to the patient's age. It also influences the quality of life. This research aims to test the null hypothesis that there is no association between the self-perceived psychosocial impacts of dental aesthetics with the severity of malocclusion, oral health-related quality of life (OHRQoL), and self-image of the body in young adults seeking orthodontic treatment.

Materials and methods: A convenience sample of young adults between 19 and 30 years old was selected for the study. The severity of malocclusion and orthodontic treatment needs were evaluated using the Dental Aesthetic Index (DAI). The Psychosocial Impact of Dental Aesthetics Questionnaire (PIDAQ), Oral Health Impact Profile-14 (OHIP-14), and Body Satisfaction Scale (BSS) were used to evaluate the self-perceived effects of malocclusion.

Statistical analysis: The Kruskal-Wallis test is used to analyze the distribution of components with different grades of DAI. Spearman's correlation test evaluated the correlation between independent variables and their domains. The study utilized stepwise multiple linear regression analysis to assess the predictive value of independent factors on the PIDAQ and its domains.

Results: A total of 181 subjects with a mean age of 24.4 ± 1.5 years, 42% males and 58% females, participated in this study. There was a significant correlation (p < 0.05) between all variables (OHIP-14, DAI, and BSS) and PIDAQ. There were significant correlations between the independent variables and the total score of PIDAQ (R2 = 0.16), psychological impact (R2 = 0.09), and social impact (R2 = 0.18), as well as dental self-confidence (R2 = 0.21) and aesthetic concern (R2 = 0.16).

Conclusion: In young adults, the self-perceived impact of dental aesthetics is moderated by the severity of malocclusion, oral health-related quality of life, and body satisfaction. The null hypothesis is rejected.

## Introduction

Malocclusion is one of the most common dental problems, characterized by an irregular arrangement of teeth or an abnormal relationship between dental arches. They significantly affect the quality of life of many people and can affect various aspects of life, including oral function, appearance, and interpersonal relationships [1].

The traditional methods used by professionals to estimate orthodontic needs or assess treatment outcomes are mainly based on normative needs (NN), which, in turn, rely on occlusal or cephalometric measurements to assess treatment outcomes [2]. They do not take into account patient-related outcomes. There is a significant difference between professional views and the patient's perception of dental appearance and the need for orthodontic treatment [3]. The patient is the best person to assess his/her health-related quality of life as it is composed of many elements of an individual's life that are not accessible to the doctor [3,4]. Patients seeking orthodontic treatment are more concerned about improving their appearance and social acceptance rather than improving their oral health and function [4]. Thus, for the assessment of orthodontic treatment needs, factors related to quality of life from the perspective of the patient and occlusal parameters from the viewpoint of the clinician must be considered.

Previous research has demonstrated a positive relationship between body image, self-esteem, self-concept, and oral aesthetics in children and adolescents [5]. The perceived influence of dental aesthetics was affected by the severity of malocclusion, the quality of life related to oral health, and happiness with one's body image [6,7]. Few studies that evaluated young adults highlighted the impact of malocclusion on oral health-related quality of life (OHRQoL) [8,9]. Most of the patients in the abovementioned studies who underwent orthodontic treatment were in the adolescent group between 12 and 18 years. However, in recent decades, the proportion of young adults seeking orthodontic treatment has increased based on their perceived aesthetic needs [10]. A review of the literature shows that studies are scarce on the psychological effects of malocclusion and hence dental aesthetics on quality of life and self-image in young adults, particularly in the Indian population. The present study aimed to evaluate, compare, and correlate the self-perceived psychosocial impact of dental aesthetics in association with malocclusion, oral health-related quality of life (OHRQoL), and body self-image in young adults seeking orthodontic treatment. A null hypothesis was put forward that there is no correlation between these factors.

## Materials and methods

Study design

The participants for this study were drawn from the outpatients registered at the Department of Orthodontics, Narayana Dental College, Nellore, India, from June 1, 2018, to May 31, 2020. Ethical approval for this cross-sectional study was obtained from the institutional ethics committee (NDC/PG/DISS/2015-2016/EC/2015, date: 16/02/2016). Participants of both genders with an age limit of 19-30 years and good physical and mental health were included. Participants with a history of trauma, craniofacial abnormalities, periodontal problems, and orthodontic treatment were excluded from the study. Informed consent was obtained from all the participants after explaining the procedure in English or their native language. Out of 202 participants who volunteered for the study, an initial evaluation to determine the severity of malocclusion was done using the Dental Aesthetic Index (DAI) [2]. Each participant was then asked to fill out the questionnaire form consisting of sociodemographic data and other three sets of questionnaires including the Psychosocial Impact of Dental Aesthetics Questionnaire (PIDAQ) [11], Oral Health Impact Profile (OHIP) [12], and Body Satisfaction Scale (BSS) [13]. In total, there were a total of 181 participants (76 males and 105 females) who returned the completed questionnaire.

Instruments used in the study

The Dental Aesthetic Index (DAI) was selected for this study due to its recognition by the World Health Organization (WHO) as a cross-cultural index [4]. The DAI is user-friendly and capable of detecting abnormal occlusal characteristics and utilizes mathematical calculations to combine clinical and aesthetic aspects into a single score. The DAI is composed of 10 distinct characteristics of malocclusion, which are assigned different weights according to their relative significance. The classification has four levels of malocclusion, each of which is associated with recommended orthodontic treatment requirements: grade 1 (DAI < 25) signifies a normal or minor malocclusion with no need for treatment or a slight need for treatment, grade 2 (26 > DAI < 30) indicates a definite malocclusion where treatment is optional, grade 3 (31 > DAI < 35) represents a severe malocclusion where treatment is highly recommended, and grade 4 (DAI < 36) corresponds to a very severe malocclusion where treatment is necessary.

The Psychosocial Impact of Dental Aesthetics Questionnaire (PIDAQ) is a 23-item psychometric tool used to evaluate certain aspects of the quality of life associated with orthodontic treatment. It measures these qualities across four areas [11]. The participants were instructed to evaluate the extent to which dental aesthetics had a favorable or unfavorable influence using a 5-point Likert scale. A higher overall score on the scale indicates a more significant influence of oral condition on the quality of life. The domains include DSC, which stands for dental self-confidence; SI, which stands for social impact; PI, which stands for psychological impact; and AC, which is for aesthetic concern. The scoring system for PIDAQ is based on a 5-point Likert scale: 0 represents the lowest level of intensity, indicating "not at all"; 1 represents a slightly higher level of intensity, indicating "a little"; 2 represents a moderate level of intensity, indicating "somewhat"; 3 represents a strong level of intensity, indicating "strongly"; and 4 represents the highest level of intensity, indicating "very strongly."

The Oral Health Impact Profile-14 (OHIP-14) is a measurement tool used to assess the impact of oral health on an individual's quality of life. The initial iteration of the OHIP-14 was employed to assess the influence of oral issues on oral health-related quality of life (OHRQoL) over the preceding six months [12]. A higher overall score on the scale indicates a more significant influence of oral condition. A 5-point scale is employed, offering the subsequent alternatives: 0 represents the frequency "never," 1 represents "hardly ever," 2 represents "occasionally," 3 represents "fairly often," and 4 represents "very often" in each of the specified categories. FL stands for functional limitation, PP represents physical pain, PD refers to psychological discomfort, PH D stands for physical disability, Ps D represents psychological disability, SD refers to social disability, and H represents handicap. The scores of each individual domain are added together to obtain a cumulative score.

The Body Satisfaction Scale(BSS) is a self-administered questionnaire used to evaluate an individual's level of contentment or discontent with 16 specific body areas. The scale consists of two components: the head parts (HP) and the overall body (BP) [13]. It has a 7-point scale to rate BSS: 1 for very satisfied, 2 for moderately satisfied, 3 for slightly satisfied, 4 for undecided, 5 for slightly unsatisfied, 6 for moderately unsatisfied, and 7 for very unsatisfied.

Statistical analysis

Statistical analysis was performed using Statistical Package for the Social Sciences (SPSS) version 21.0 (IBM SPSS Statistics, Armonk, NY). Cronbach's alpha coefficient was used to measure the internal consistency or reliability of the self-administered score. The data assessed by the Shapiro-Wilk test indicated that the distribution of values is non-parametric. The Kruskal-Wallis test was used to compare variables associated with different grades of DAI, and the Mann-Whitney test was used for the comparison of intrapair differences. The bivariate correlation between all continuous variables was evaluated using Spearman's correlation test. Stepwise multiple linear regression analysis was used to evaluate the predictable value of independent variables of the study with PIDAQ and its domains. The level of significance was set at p < 0.05 for all tests.

## Results

A total of 181 participants (76 (42%) males and 105 females (58%)) were included in this study. The age of the participants ranged from 19 to 28 years (24.4 ± 1.5 years). The descriptive variables of clinical assessment (DAI) and the scores of the self-administered questionnaires of the study (PIDAQ, OHIP-14, and BSS) were given in Table 1. We found an intra-class correlation coefficient (ICC) of 98% for DAI, measured at a one-week interval. The Cronbach's alpha coefficient values ranged from 0.92 for PIDAQ (subscale values: 0.88-0.92), 0.90 for OHIP-14, and 0.92 for BSS. Thus, the scores showed high internal consistency and reliability. Most of them had grade 1 severity (58%), with no need or slight need for treatment. We combined grade 3 and grade 4 for statistical purposes since only one of the participants had grade 4 needs.

**Table 1:** Clinical and psychosocial characteristics of the participants of the study *p < 0.05: statistically significant, p > 0.05: statistically not significant DAI: Dental Aesthetic Index, SD: standard deviation, PIDAQ: Psychosocial Impact of Dental Aesthetics Questionnaire, DSC: dental self-confidence, SI: social impact, PI: psychological impact, AC: aesthetic concern, OHIP-14: Oral Health Impact Profile-14, FL: functional limitation, PP: physical pain, PD: psychological discomfort, PH D: physical disability, PS D: psychological disability, SD: social disability, H: handicap, BSS: Body Satisfaction Scale, HP: head parts, BP: body parts

	Number	Possible total score	Mean (SD)	Median	Minimum-maximum
I. DAI					
Grade 1	106	13-25	20.72 (2.73)	21	17-25
Grade 2	58	25-30	27.43 (1.36)	27	26-30
Grade 3	16	30-35	33.18 (1.18)	34	31-35
Grade 4	1	>36	43	43	36-43
Grade 3 + 4	17		33.75 (6.5)	34	31-43
DAI total	181		24.09 (4.95)	24	17-43
II. PIDAQ					
DSC	181	0-18	12.06 (5.43)	12	1-24
SI	181	0-24	6.82 (5.01)	6	0-19
PI	181	0-18	6.30 (4.34)	6	0-22
AC	181	0-9	2.08 (3.0)	0	0-12
PIDAQ total	181	0-69	27.28 (10.37)	24	5-47
III. OHIP-14					
FL	181	0-8	0.66 (1.22)	0	0-4
PP	181	0-8	1.10 (1.46)	0	0-5
PD	181	0-8	1.34 (1.64)	1	0-6
PH D	181	0-8	0.60 (1.28)	0	0-5
PS D	181	0-8	0.8 (1.23)	0	0-6
SD	181	0-8	0.69 (1.33)	0	0-4
H	181	0-8	1.08 (1.31)	1	0-5
OHIP-14 total	181	0-56	6.3 (6.97)	4	0-28
IV. BSS					
HP	181	16-112	13.36 (6.75)	11	8-32
BP	181	8-56	11.35 (5.33)	9	8-36
BSS total	181	8-56	24.71 (11.05)	21	16-67

Among all the individuals, 98.3% demonstrated a psychological impact associated with dental aesthetics. Additionally, 92% of the participants reported experiencing at least one oral influence on their quality of life. When comparing the overall scores, the BSS had the greatest mean score of 24.71 ± 10.37 out of a total score of 56, surpassing both OHIP-14 and PIDAQ. Of the samples, 82% expressed dissatisfaction with some bodily parts, with the head component scoring 13.36 ± 6.75, while the total bodily scores were 11.35 ± 5.33 (Table 1).

Table 2 shows the distribution of the total PIDAQ, OHIP-14, BSS, and their subcomponents within each grade of DAI. In general, the PIDAQ scale and its subscales are directly proportional to the DAI scores. Dental self-confidence (DSC) has the maximum psychosocial impact, and it increases with the severity of DAI. The total PIDAQ scores (p = 0.02) and the DSC domain PIDAQ scores (p < 0.001) showed that there was a statistically significant difference between the DAI grades. However, there was no statistically significant difference between the grades in the scores for the other scales or subscales.

**Table 2:** Means and standard deviations of PIDAQ scale, OHIP-14 scale, BSS, and subscales according to DAI grades of malocclusion (Kruskal-Wallis test) *p < 0.05: statistically significant, p > 0.05: statistically not significant a, b, and c: significant difference between the groups PIDAQ: Psychosocial Impact of Dental Aesthetics Questionnaire, OHIP-14: Oral Health Impact Profile-14, BSS: Body Satisfaction Scale, DAI: Dental Aesthetic Index, SD: standard deviation, DSC: dental self-confidence, SI: social impact, PI: psychological impact, AC: aesthetic concern, FL: functional limitation, PP: physical pain, PD: psychological discomfort, PH D: physical disability, PS D: psychological disability, SD: social disability, H: handicap, HP: head parts, BP: body parts

Other scales	DAI grade						Chi-square value	p-value
	1 (n = 106)		2 (n = 58)		3+4 (n = 17)			
	Mean (SD)	Median	Mean (SD)	Median	Mean (SD)	Median		
DSC	10.4 (5.10)^a^	9	13.67 (4.45)^b^	14	16.94 (6.26)^c^	20	28.15	0.001*
SI	6.97 (5.27)	6	6.53 (4.81)	6	6.94 (4.41)	8	0.30	0.86
PI	6.3 (4.25)	6	6.31 (4.57)	6	6.29 (4.59)	8	0.08	0.96
AC	2.41 (3.29)	1	1.66 (1.72)	0	1.59 (2.69)	0	1.36	0.51
PIDAQ total	26.08 (10.71)^a^	23	28.17 (10.05)^b^	26.5	31.76 (8.42)^c^	31	7.79	0.02*
FL	0.63 (1.30)	0	0.72 (1.18)	0	0.65 (0.86)	0	2.15	0.34
PP	1.05 (1.48)	0	1.24 (1.51)	1	1 (1.28)	1	1.03	0.60
PD	1.17 (1.65)	0	1.57 (1.56)	1	1.71 (1.86)	1	5.85	0.06
PH D	0.59 (1.29)	0	0.78 (1.43)	0	0.06 (0.24)	0	3.77	0.15
PS D	0.83 (1.27)	0	0.9 (1.31)	0	0.35 (0.70)	0	2.95	0.23
SD	0.75 (1.42)	0	0.69 (1.33)	0	0.41 (0.80)	0	0.29	0.87
H	1.18 (1.34)	1	1.09 (1.41)	1	0.47 (0.51)	0	3.79	0.15
OHIP-14 total	6.2 (7.52)	4	6.98 (6.73)	5.5	4.65 (3.53)	3	3.17	0.21
HP	13.88 (7.35)	11	12.1 (5.09)	10	14.47 (7.84)	12	0.86	0.65
BP	11.52 (5.55)	9	10.48 (3.43)	8.5	13.29 (8.42)	10	0.60	0.74
BSS total	25.4 (12.08)	21	22.59 (6.53)	21	27.76 (15.62)	24	0.22	0.90

The cross-tabulation of the correlation between the total scores and subscores of all domains of DAI, PIDAQ, OHIP-14, and BSS is shown in Table 3. DAI shows a strong positive correlation with DSC (p = 0.27), which is significant. DSC shows a strong positive correlation with the domains of FL (r = 0.21), PP (r = 0.16), and PD (r = 0.17) of the OHIP-14 score, which is statistically significant. A strong negative correlation was found with the total scores of BSS (r = -0.23) and its subscores HP (r = -0.23), and BP (r = -0.16).

**Table 3:** Correlation coefficient for the analysis of the association between DAI scores and components of other scales (PIDAQ, OHIP-14, and BSS) (Spearman correlation coefficient) *p < 0.05: statistically significant, p > 0.05: statistically not significant, rs values: Spearman correlation DAI: Dental Aesthetic Index, PIDAQ: Psychosocial Impact of Dental Aesthetics Questionnaire, OHIP-14: Oral Health Impact Profile-14, BSS: Body Satisfaction Scale, DSC: dental self-confidence, SI: social impact, PI: psychological impact, AC: aesthetic concern, HP: head parts, BP: body parts, FL: functional limitation, PP: physical pain, PD: psychological discomfort, PH D: physical disability, PS D: psychological disability, SD: social disability, H: handicap

Spearman correlation		DAI score	OHIP-14 total	PIDAQ total	BSS total	PIDAQ components				BSS components	
						DSC	SI	PI	AC	HP	BP
		r_s _values	r_s _values	r_s _values	r_s _values	r_s _values	r_s _values	r_s _values	r_s _values	r_s _values	r_s _values
Total indices	DAI score	1.00	0.06	0.14	0.00	0.27*	0.02	0.02	-0.10	-0.04	-0.02
	OHIP-14 total	0.06	1.00	0.24*	0.35*	0.02	0.28*	-0.24*	0.28*	0.37*	0.20*
	PIDAQ total	0.14	0.24*	1.00	0.01	-	-	-	-	0.03	0.04
	BSS total	0.00	0.35*	0.01	1.00	-0.23*	0.12	0.09	0.18*	-	-
PIDAQ components	DSC	0.27*	0.02	-	-0.23*	1.00	-	-	-	-0.23*	-0.16*
	SI	0.02	0.28*	-	0.12	-	1.00	-	-	0.14	0.10
	PI	0.02	0.24*	-	0.09	-	-	1.00	-	0.10	0.09
	AC	-0.10	0.28*	-	0.18*	-	-	-	1.00	0.21*	0.19*
OHIP-14 components	FL	0.07	-	0.35*	0.04	0.21*	0.28*	0.21*	0.21*	0.06	0.04
	PP	0.03	-	0.19*	0.15*	0.16*	0.24*	0.12	0.24*	0.15	0.12
	PD	0.13	-	0.24*	0.10	0.17*	0.13	0.26*	0.23*	0.13	0.04
	PH D	-0.03	-	0.22*	-0.01	-0.06	0.20*	0.27*	0.31*	0.06	-0.07
	PS D	-0.07	-	0.20*	0.15*	-0.96^*^	0.25*	0.32*	0.31*	0.18*	0.06
	SD	0.00	-	0.13	0.16*	-0.09	0.11*	0.23*	0.08	0.19*	0.03
	H	-0.10	-	0.14	0.09	-0.18	0.33*	0.17*	0.26*	0.17*	-0.09
BSS components	HP	-0.04	0.37*	0.03	-	-0.23*	0.14	0.10	0.21*	1.00	-
	BP	-0.02	0.20*	0.04	-	-0.16*	0.10	0.09	0.19*	-	1.00

The multiple linear regression analysis showed that the independent variables (BSS, OHIP-14, and DAI score) had a large effect on how patients felt about the psychological and social effects of dental aesthetics. The data presented in Table 4 support this finding. The regression models used age as a controlled variable. The R2 values for the whole of the PIDAQ scale and its subscales indicate that the model explains 9%-21% of the variability in perception scores.

**Table 4:** Multiple linear regression for the association of PIDAQ and its components with independent variables *p < 0.05: statistically significant, p > 0.05: statistically not significant PIDAQ: Psychosocial Impact of Dental Aesthetics Questionnaire, DSC: dental self-confidence, SI: social impact, PI: psychological impact, AC: aesthetic concern, DAI: Dental Aesthetic Index, OHIP-14: Oral Health Impact Profile-14, BSS: Body Satisfaction Scale

Regression parameter	Independent variable	Dependent variable				
		PIDAQ total	DSC	SI	PI	AC
Beta coefficient	(Constant)	19.28*	12.54	3.77	2.11	0.87
	Age (years)	-0.514	-0.288	-0.124	-0.248	-0.044
	Sex (male (0))	-2.11	-2.71*	-0.62	1.20	0.01
	DAI score	0.25	0.29*	0.01	0.01	-0.06
	OHIP-14 total	0.51*	-0.06	0.27*	0.19*	0.12*
	BSS total	0.07	-0.13*	0.08*	0.04*	0.08*
F		8.63	11.34	9.90	4.47	8.57
R^2^		0.16	0.21	0.18	0.09	0.16
p		<0.001*	<0.001*	<0.001*	0.002*	<0.001*

## Discussion

Dental aesthetics due to malocclusion can have a negative impact on quality of life, social engagement, community ties, and psychological well-being. Helm et al. [14], in their longitudinal study, found that malocclusion plays an important role in general body image, and it may affect self-concept not only in adolescence but also in adulthood. Appearance dissatisfaction during adolescence can lead to depression, loneliness, and low self-esteem, which can continue into early adulthood, leading to passive social interaction and behavior [15]. It is better to analyze the self-perceived psychosocial impact of dental aesthetics in adults, as they already possess a certain emotional stability and have a more realistic view of dentofacial aesthetics [1,9]. This study was taken up to explore if there is a considerable difference between patients' and professional assessment of orthodontic treatment needs and whether there is a relation between the severity of malocclusion and the self-perceived impact of it on the quality of life.

The normative treatment need was assessed using the Dental Aesthetic Index (DAI). The current study had a relatively smaller number of subjects in the highly desirable and definite treatment need categories. The self-rating instrument PIDAQ was designed to assess the psychosocial impact of dental aesthetics and, hence, the impact of oral health-related quality of life in young adults. The individuals with no or minimal malocclusion showed lower PIDAQ scores than individuals with malocclusion. PIDAQ increased with an increase in DAI grade.

**Figure 1:**
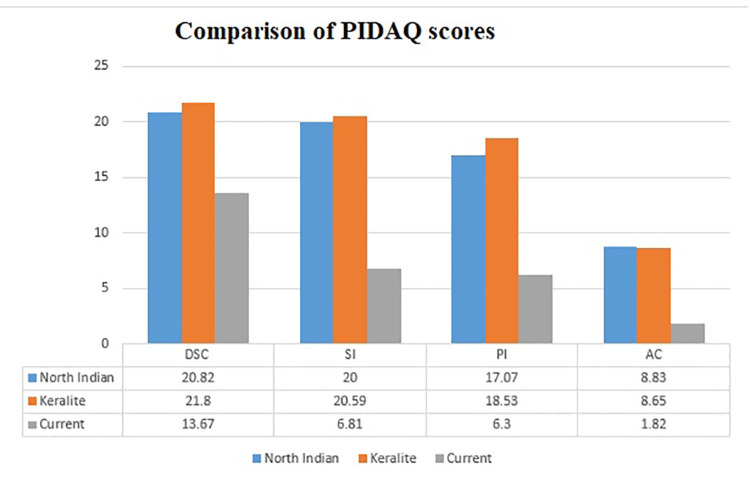
Graph showing the comparison of mean values of PI, AC, SI, and DSC among earlier Indian studies and the present study PIDAQ: Psychosocial Impact of Dental Aesthetics Questionnaire, PI: psychological impact, AC: aesthetic concern, SI: social impact, DSC: dental self-confidence

Dental self-confidence (DSC) measures the influence of dental aesthetics on the self-image of an individual, and it indicates the level of satisfaction with one's own dental appearance [8]. There is decreased self-confidence with an increase in DAI scores. What was unique about PIDAQ was its attempt to incorporate DSC, a measure of positive characteristics based on one's teeth. Because the items in this area were reverse-scored, a high score suggests that subjects had a low level of self-confidence based on their dental appearance and vice versa. Dental self-confidence is a positive sense of well-being related to one's own dental alignment, and the scores increased significantly with the severity of malocclusion. Thus, it reflected the convergence between normative and psychometric assessments of malocclusion (Table 1). The other individual domains of PIDAQ such as social impact (SI), psychosocial impact (PI), and aesthetic concern( AC) are not significantly associated with the severity of malocclusion, except for the subscores of dental self-confidence (DSC) (Table 1 and Table 2). The subscales of PIDAQ of the current study showed a level of concern much less compared to that of the North Indian [16] and Keralite [17] populations that applied the Hindi and Malayalam versions of the PIDAQ questionnaire, demonstrating regional differences (Figure 1). The OHIP-14 scores (6.98) reported in this study were also less than those of a previous study by Masood et al. [18] on young adults (22.6). The differences in the scores might be in both the perception of malocclusion and the interpretation of OHRQoL due to cultural variations. The psychological discomfort domain (1.35) shows the highest impact in the current study, whereas the physical disability (0.6) in adults has the least. Mahmood and Kareem [19], in their study conducted on young adults, concluded that orthodontic patients mainly suffer from aesthetic and social problems rather than impairment of daily activities.

The total PIDAQ scores and its components are significantly correlated with the other scores of the study (Table 3). The interrelation between the other scores and subscores is inconsistent. This is in partial concordance with the earlier study of de Paula et al. [7], who reported that there was no association between OHIP, DAI, and BSS, as these were not designed specifically to measure the effects of orthodontic problems alone and also pose some questions that are not always relevant to the patients with malalignment of teeth. We found the correlation between any two measured parameters to be within 0.3, indicating a weak correlation. The results are in tune with the study of Figueroa et al. [20]. All the indices for determining orthodontic treatment needs, including DAI, have an aesthetic component, but they are based on occlusion and functional features that do not accurately represent the perceptions of the patient. They stated that beauty is a completely subjective concept, and only malocclusion may not affect self-perception negatively.

In the current study, DSC demonstrated the highest predictive value and the least psychological impact (Table 4). In adults, gender and DAI are significant predictors of DSC. There is a significant gender difference in normative needs, and females present with a greater impact than males. The BSS shows a significant predictive value for DSC, social impact, and aesthetic concern in adults. As facial aesthetics is influenced by dental appearance, the facial image is more associated with self-perceived psychosocial impact. We found that the severity of malocclusion, quality of life in relation to oral health, and body self-image all moderated young adults' self-perceived impact of dental aesthetics, and hence, the null hypothesis is rejected.

We conducted the first study of its kind on the Indian population of young adults, assessing the difference between normative and perceived needs based on the severity of malocclusion. Ours is also the first study that seeks to correlate the self-perceived psycho-sociological impacts of dental aesthetics with their effects on quality of life. No study goes without limitations. One of the main limitations of the study is the small sample size. Given the number of items on the PIDAQ scale, a minimum of 230 subjects would have been required [17]. We used the PIDAQ in its original English version. A validated version in the native language would have been better. Furthermore, the convenience sample in this study may not truly represent the population percentage. This study did not differentiate between the different types of malocclusion. A long-term study that looks at specific malocclusions would show the true treatment needs and what psychological effects the aesthetic component has. While treating the malocclusion, the orthodontist should keep in mind the patient's gender and age, give importance to the patient's perceived needs, and satisfy their expectations within the possible limits of the treatment plan.

## Conclusions

There is a significant variation in some subscales and domains of the subjective self-perception of dental aesthetics. There is a significant association between psychosocial impact and malocclusion, but the correlation is not strong. Occlusal disorders, quality of life in relation to oral health, and self-image are typical factors that impact how one perceives oneself. When taken as a whole, these metrics can provide a solid picture of where to direct treatment efforts.
